# Analysis of gene duplication within the Arabidopsis NUCLEAR FACTOR Y, subunit B (NF-YB) protein family reveals domains under both purifying and diversifying selection

**DOI:** 10.1371/journal.pone.0289332

**Published:** 2023-08-02

**Authors:** Chamindika L. Siriwardana, Jan R. Risinger, Emily Mills Carpenter, Ben F. Holt

**Affiliations:** 1 Department of Microbiology and Plant Biology, University of Oklahoma, Norman, Oklahoma, United States of America; 2 Department of Science and Mathematics, Texas A&M University-Central Texas, Killeen, Texas, United States of America; 3 Myriad Genetics Corporation, Salt Lake City, Utah, United States of America; 4 Aquatic Biomonitoring, Austin, Texas, United States of America; 5 AgBiome, Research Triangle Park, North Carolina, United States of America; Università degli Studi di Milano, ITALY

## Abstract

Gene duplication is an evolutionary mechanism that provides new genetic material. Since gene duplication is a major driver for molecular evolution, examining the fate of duplicated genes is an area of active research. The fate of duplicated genes can include loss, subfunctionalization, and neofunctionalization. In this manuscript, we chose to experimentally study the fate of duplicated genes using the Arabidopsis NUCLEAR FACTOR Y (NF-Y) transcription factor family. NF-Y transcription factors are heterotrimeric complexes, composed of NF-YA, NF-YB, and NF-YC. NF-YA subunits are responsible for nucleotide-specific binding to a *CCAAT* cis-regulatory element. NF-YB and NF-YC subunits make less specific, but essential complex-stabilizing contacts with the DNA flanking the core *CCAAT* pentamer. While ubiquitous in eukaryotes, each NF-Y family has expanded by duplication in the plant lineage. For example, the model plant Arabidopsis contains 10 each of the NF-Y subunits. Here we examine the fate of duplicated NF-YB proteins in Arabidopsis, which are composed of central histone fold domains (HFD) and less conserved flanking regions (N- and C-termini). Specifically, the principal question we wished to address in this manuscript was to what extent can the 10 Arabidopsis NF-YB paralogs functionally substitute the genes *NF-YB2* and *NF-YB3* in the promotion of photoperiodic flowering? Our results demonstrate that the conserved histone fold domains (HFD) may be under pressure for purifying (negative) selection, while the non-conserved N- and C-termini may be under pressure for diversifying (positive) selection, which explained each paralog’s ability to substitute. In conclusion, our data demonstrate that the N- and C-termini may have allowed the duplicated genes to undergo functional diversification, allowing the retention of the duplicated genes.

## Introduction

Gene duplication is a major driver of molecular evolution [1]. Most genes found in all forms of life—bacteria, archaebacteria, and eukaryotes—have been generated by gene duplication events [2]. The fate of duplicated genes has been an area of intense research since the publication of Evolution by gene duplication by Dr. Susumu Ohno in 1970. Broadly, the fate of the duplicated gene copies can be one of three fates: loss, subfunctionalization (mutations affect different functions of each copy, such that both copies are required to preserve all ancestral gene functions [1, 3] and neofunctionalization (one copy retains its ancestral functions, and the other acquires a novel function) [1, 4]. The most likely fate of gene duplication is loss. However, certain classes of genes appear to be preferentially retained, such as those encoding transcription factors [5–7]. Duplicates that are retained can undergo subfunctionalization or neofunctionalization. In eukaryotes, it is estimated that plants have undergone more frequent gene duplication events than animals, leading to higher genome diversity. Genome diversity in the plant lineage includes polyploidy, with estimates of polyploidy in angiosperms varying between 30 to 80% [8]. It is also assumed that many plant species that are diploid today were paleopolyploid, containing polyploid ancestors [9].

The model plant *Arabidopsis thaliana* (Arabidopsis), a diploid species, is predicted to have undergone at least two independent whole genome duplication (WGD) events [10]. In addition, Arabidopsis is predicted to have undergone several segmental and single-gene duplication events [11]. The duplicated gene content in Arabidopsis is estimated to be between 47% to 63%. This includes large gene families such as receptor kinases with an estimate of ~ 400 members and cytochrome P450 genes with an estimate of ~ 270–285 members and many smaller gene families [9, 12]. An example of subfunctionalization or neofunctionalization of duplicated genes is the observation that about 75% of duplicated genes have shown divergent expression patterns in microarray analysis [5]. However, many of the examples have focused on functional conservation across orthologs, and much fewer between whole families of paralogs [13–16]. Here we attempt to experimentally demonstrate the fate of duplicated genes by studying a family of paralogs from Arabidopsis, the Nuclear Factor Y (NF-Y).

NF-Y is a heterotrimeric transcription factor family ubiquitous to eukaryotes. Three independent subunits NF-YA, NF-YB, and NF-YC form the mature transcription factor complex that binds DNA at site-specific *CCAAT* boxes. Animals, including humans and mice, have one each of an NF-YA, NF-YB, and NF-YC subunit [17]. In contrast, the NF-Y has undergone extensive expansion in the plant lineage. For example, Arabidopsis contains 10 subunits each of NF-YA, NF-YB, and NF-YC [18]. The expansion holds for other plant species, including monocots and dicots. The structure of each subunit required for DNA contact trimer formation has been extensively studied in animal models. All three subunits are required for *CCAAT* box binding, with the NF-YA subunit making specific contacts with the *CCAAT* box and the NF-YB and NF-YC subunits stabilizing the complex [19–21]. The NF-YB and NF-YC subunits contain a histone fold domain (HFD) and mimic H2A/H2B-DNA binding during its association with DNA [22]. These subunits have evolved from core histone proteins and are predicted to contain similar three-dimensional confirmations [23]. The 3D structure of the HFD is thought to contain three helices (alpha 1, 2, and 3), which are separated by two loops (L1 and L2). Analysis of the crystal structure has shown that the HFD which spans a region of approximately 96 amino acids, is required for trimer formation and DNA contacts [22].

Functional analysis in animals has demonstrated that the HFD alone can function in protein-protein and protein-DNA interactions [19, 24]. While the HFD is highly conserved, the HFD is flanked by a non-conserved N- and C-termini. In Arabidopsis, the NF-YB and NF-YC subunits only require the HFD for protein-protein interactions with each other (this paper). However, the Arabidopsis NF-Y has been shown to require components of the N- and C-termini for interaction with non-NF-Y proteins [25]. Extensive tissue-specific expression analysis on the Arabidopsis NF-Y subunits has demonstrated that each subunit has specific temporal and special expression patterns [18]. For example, when comparing the 10 NF-YB subunits, NF-YB2, NF-YB3, and NF-YB7 are the only subunits that have a strong expression in 10-day-old rosettes. The expression pattern is consistent with their function, where these three subunits have a demonstrated role in regulating flowering responses ([26] and this paper).

The principal question we wished to address in this manuscript was to what extent can the 10 Arabidopsis NF-YB paralogs functionally substitute for each other in the promotion of photoperiodic flowering. In other words, we know that they vary significantly in 1) tissue- and developmental-specific expression patterns and 2) protein sequence composition, but we do not know to what extent this variability results in changes in functional capacity. Previous research has demonstrated that apparent orthologs from other species can readily substitute for Arabidopsis NF-Y proteins [27]. However, studies within a species to study the paralogs have not been done. Therefore, here we use an analysis of paralogs within a single species to begin to address this question.

## Results

### Based on the phylogenetic relationships, the NF-YB family members can be classified into four ancestrally related sub-classes

**Fig 1:**
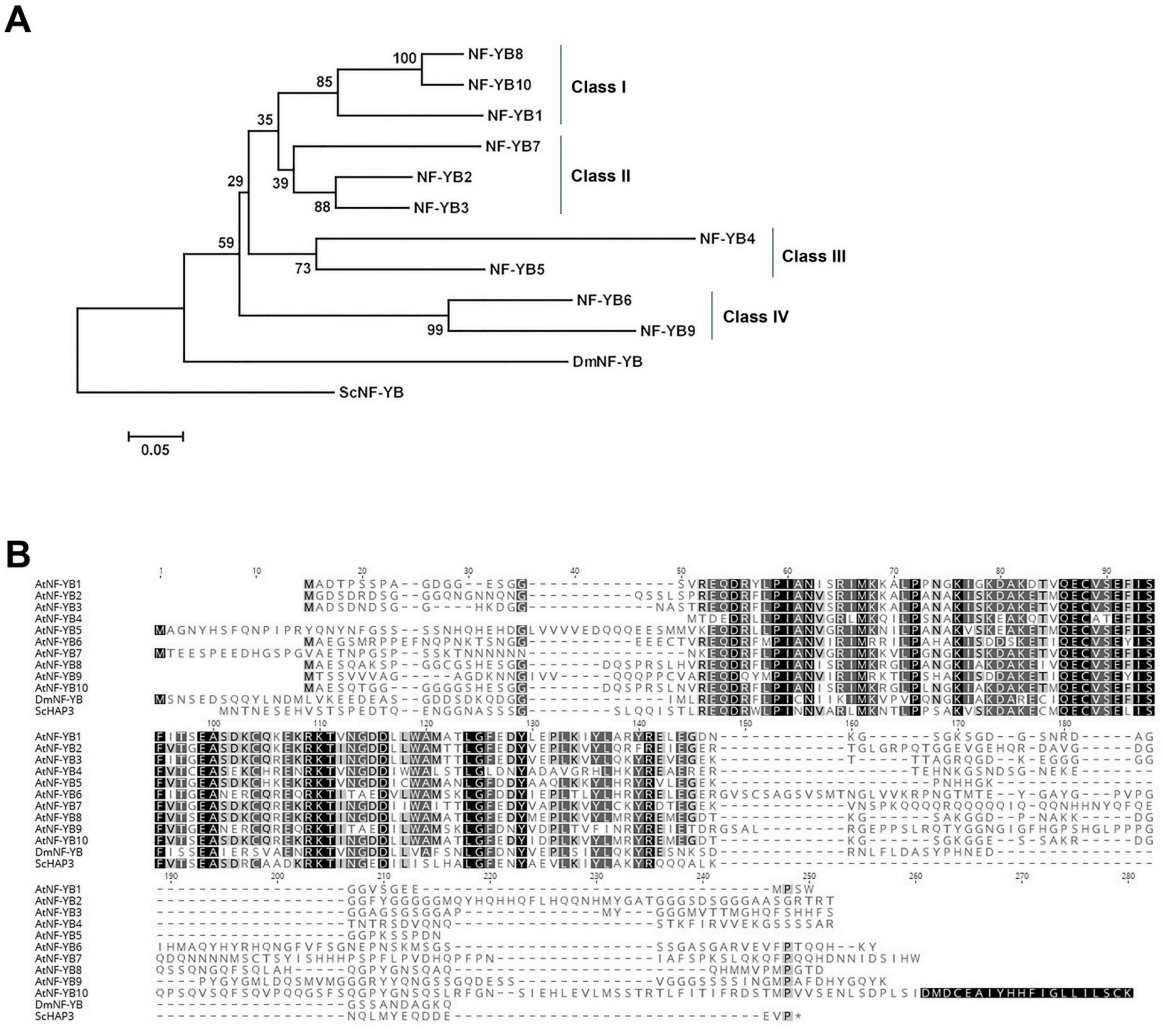
Arabidopsis NF-YB proteins are grouped into four distinct clades. A) Neighbor-joining phylogenetic tree of NF-YB full-length proteins constructed in MEGA7. Reliability values at each branch represent bootstrap samples with 1000 replicates. B) Multiple Sequence Alignment (MSA) of the Arabidopsis NF-YB full-length proteins. The MSA was constructed using MUSCLE within Geneious. Sc, *Saccharomyces cerevisiae* and Dm, *Drosophila melanogaster*.

We examined the phylogenetic relationships of the NF-YB family members using both neighbor-joining (NJ) and maximum-likelihood (ML) methods for full-length proteins, HFD regions alone, and the nucleic acid sequence of the coding regions (Fig 1A and S1 Fig). NJ and ML methods gave largely similar trees, with eight of the 10 proteins/genes (depending on tree type) showing clear and consistent pairings to a recently diverged paralog. NF-YB1 did not have such an obvious pairing, but all trees suggest that it most recently shared an ancestor with NF-YB8 and NF-YB10. The placement of NF-YB7 in the various trees was consistently the least reliable. In some trees, it appears to share a recent ancestor with NF-YB2 and NF-YB3, while in others that relationship maps to NF-YB4 and NF-YB5. However, in all cases, the bootstrap values were well below 70% and not reliable for accurately inferring evolutionary relationships. We further extended the phylogenetic analysis by including monocot and dicot species in which the NF-Y has been identified and characterized; *Brachypodium distachyon*, *Triticum aestivum*, *Citrus sinensis*, and *Prunus Persia* [27–30]. The Arabidopsis NF-Y showed a similar pairing as earlier when the tree included other plant species; NF-YB1, NF-YB8, and NF-YB10; NF-YB2, NF-YB3, and NF-YB7; NF-YB4, and NF-YB5; NF-YB6 and NF-YB9 were placed in the same clade respectively (S2 Fig). The orthologs for the Arabidopsis NF-Y have been previously identifies in *Brachypodium distachyon*, *Citrus sinensis*, and *Prunus Persia* [27–29]. In Arabidopsis NF-YB6 and NF-YB9, two paralogs with clear and consistent paring, which are functionally conserved and are involved in embryo development [18] share the putative Brachypodium orthologs BdNF-YB2, BdNF-YB4 and BdNF-YB17. When we study the multispecies phylogenetic tree (S2 Fig), the Arabidopsis NF-YB6, NF-YB9 and the Brachypodium BdNF-YB2, BdNF-YB4 and BdNF-YB17 fall into the same clade. No other subunits from Arabidopsis or Brachypodium fall on to this clade. The same is true for Prunus PpNF-YB4, PpNF-YB7, and PpNF-YB8 and Citrus CsL1L-1, CsL1L-2, and CsLEC1. The placement of the other orthologs had similar patterns but were not as strongly supported. For example, the placement of NF-YB2, NF-YB3 and NF-YB7 together again was less clear with the placement of the orthologs. The orthologs of NF-YB2 and NF-YB3; BdNF-YB1, BdNF-YB6 BdNF-YB12, PpNF-YB3, PpNF-YB5, PpNF-YB6 and PpNF-YB9, CsNF-YB3 were in the same clade. However, the closest ortholog to NF-YB7, CsNF-YB7 was in the same clade as NF-YB1, NF-YB8, and NFYB10.

Based on the phylogenetic relationships we grouped the NF-YB family members into four ancestrally related sub-classes; Class I–NF-YB1, NF-YB8, and NF-YB10; Class II–NF-YB2, NF-YB3, and NF-YB7; Class III–NF-YB4 and NF-YB5; Class IV–NF-YB6 and NF-YB9. Although the ancestral path to NF-YB7 was difficult to predict, the choice to place it in Class II was based on its overall higher percentage similarity to NF-YB2 and NF-YB3 than other proteins (S3 Fig). Perhaps more relevant, we used flowering time assays to clarify these possible functional relationships (see below).

### NF-YB proteins show a high degree of conservation in their shared histone fold domains (HFD), flanked by non-conserved N- and C-termini

Initially, we examined alignments of all 10 Arabidopsis NF-YB paralogs, as well as their singular orthologs in *Saccharomyces cerevisiae* (ScNF-YB) and *Drosophila melanogaster* (DmNF-YB). Alignment of the amino acid sequences revealed a high degree of conservation in their central HFDs ([31, 32] and Fig 1B). Within Arabidopsis, the identity in pairwise comparisons—confined to only the HFD—ranged from 53 to 95%, with a mean identity/similarity of 71/93% (S3 Fig). When making pairwise comparisons, conservation decreases precipitously in the N- and C-termini flanking the central HFD. Depending on the protein, the N-terminus spans 20–50 amino acids (aa), except for NF-YB4, which lacks an N-terminus and the first four amino acids of the HFD. The length of the C-terminus varies from 15 aa in NF-YB5 to 104 aa in NF-YB10. To further evaluate the N- and C-termini we constructed individual alignments of the four ancestrally related sub-classes (S4 and S5 Figs). The alignments show conservation across subclasses, especially between the N termini of subclasses I, II and IV and the C termini of subclasses I, III, and IV.

### NF-YB paralogs can both positively and negatively alter photoperiod-dependent flowering time

From both loss-of-function and overexpression analyses, we know that *NF-YB2* and *NF-YB3* are required, positive regulators of photoperiod-dependent flowering [26, 33–36]. While expression analyses clearly show that NF-YB paralogs have highly variable tissue- and development-specific expression patterns, the extent to which their encoded proteins have functionally diversified remains unknown. Therefore, we initially examined the capacity of each NF-YB to alter flowering time when overexpressed (35S cauliflower mosaic virus promoter [37]) in the wild-type Col-0 background. For each overexpressed gene, we examined the flowering time for pools of independent, first-generation (T1) plants–a method we have previously successfully used and justified [38]. In short, observations of numerous T1 plants avoid observer bias and correct for positional effects of independent transgenic insertion events. Phenotyping T1 plants also gives an accurate view of the variability associated with each overexpressed gene.

**Fig 2:**
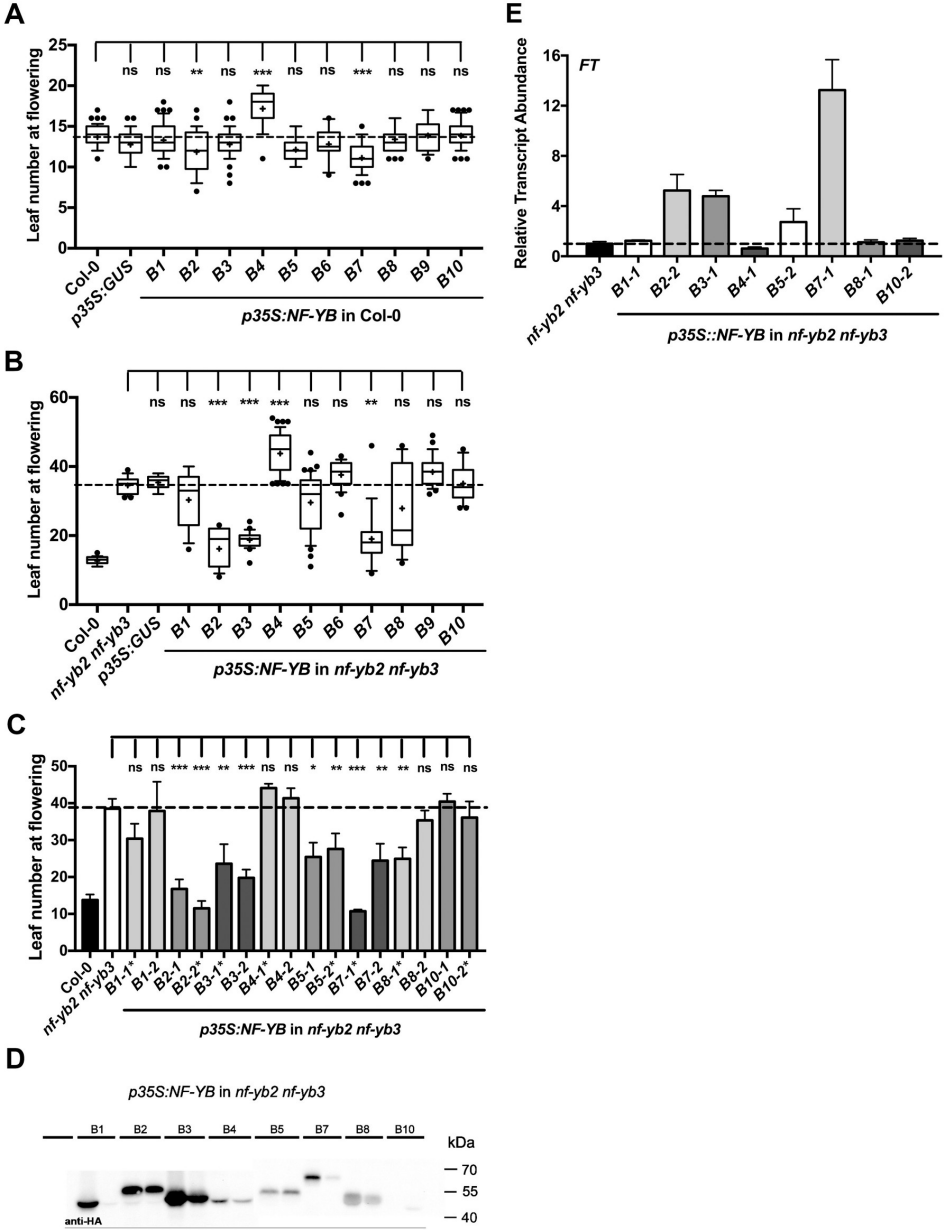
T1 flowering time quantification of full-length *p35S*:*NF-YB*:*YFP*:*HA* constructs in A) Col-0 background B) *nf-yb2 nf-yb3* background. C) Flowering time quantifications of two independent (line 1 and line 2) stable T3 generation full-length *35S*:*NF-YB*:*YFP*:*HA* constructs in the *nf-yb2 nf-yb3* background. Asterisks* Represent the stable line used in 2E and S3A and S3B Fig. D) Protein expression levels of each of the lines assayed in (C). E) qRT-PCR analysis of *FT* mRNA levels of stable T3 generation *35S*:*NF-YB*:*YFP*:*HA* lines in the *nf-yb2 nf-yb3* background. In (A) and (B) cross represents the mean, and outliers represent data points <10th and >90th percentile, respectively. Approximately 20 independent T1 plants were examined for each gene and each experiment was repeated with similar results. Asterisks represent significant differences derived by the Kruskal-Wallis test (P < 0.05) followed by Dunn’s multiple comparisons posthoc test against Col-0 (A) or *nf-yb2 nf-yb3* (B and C) (* P<0.05, ** P<0.01, *** P<0.001).

*NF-YB2* and *NF-YB7* overexpression resulted in early flowering by approximately 2–3 leaves, while the other Class II gene, *NF-YB3*, did not significantly alter flowering time. However, we note that *NF-YB3* overexpression resulted in a trend towards earlier flowering, consistent with its known role as a positive regulator of flowering from loss of function analyses [26] (also see below). Alternatively, overexpression of *NF-YB4* consistently gave significantly later flowering than parental Col-0 (Fig 2A). As NF-YB4 protein does not have an extended N terminus and is missing the first four amino acids of the HFD, this suggests it may be acting as a dominant negative by unproductively interfering with flower-promoting NF-Y complexes.

While the initial overexpression analyses in Col-0 did provide new information regarding *NF-YB4* and *NF-YB7*, the analyses suffered from the disadvantage that known flower-promoting *NF-YB2* and *NF-YB3* were still accumulating in these otherwise wild-type transgenic plants. We reasoned that overexpressing the same suite of 10 *NF-YB* genes in the late flowering *nf-yb2 nf-yb3* double mutant would allow us to more effectively quantitate the relative capacity of each gene to regulate photoperiod-dependent flowering. An alternative approach to overexpression would have been to express each test *NF-YB* from either the *NF-YB2* or *NF-YB3* promoter. Unfortunately, this would preclude T1 experiments because heterozygous expression of either *NF-YB2* or *NF-YB3* in the *nf-yb2 nf-yb3* background does not significantly alter the late flowering phenotype of the double mutant [26]. Results show that the wild-type Col-0 and mutant *nf-yb2 nf-yb3* flowered at 12.7 (±1.1 stdev) and 34.5 (±2.5) leaves, respectively (Fig 2B). As expected, overexpression of *NF-YB2* or *NF-YB3* in the double mutant background resulted in near complete rescue of the late flowering phenotype at 16.2 (±5.5) and 18.7 (±2.3) leaves, respectively. Once again, overexpression of the other Class II gene, *NF-YB7*, also resulted in earlier flowering at 19.0 (±8.2) leaves, supporting the phylogenetic and functional clustering of these three genes (Fig 1). No other gene drove significantly earlier flowering on average. However, overexpression of *NF-YB1* (Class I), *NF-YB5* (Class III), and *NF-YB8* (Class I) showed trends towards earlier flowering, and plants with higher accumulation of their respective proteins were consistently associated with earlier flowering (see below).

To further investigate the relationship between NF-YB protein accumulation and flowering time in the *nf-yb2 nf-yb3* background, we isolated two stable, single-insertion T3 transgenic plant lines for each overexpressed *NF-YB* gene, except for NF-YB6 and NF-YB9 (Fig 2C and 2D). We were unable to construct stable T3 generation plant lines for *p35S*:*NF-YB6* and *p35S*:*NF-YB9* and did not use these lines for further analysis. Most of these individuals were initially chosen because their T1 parental lines flowered at different times (earlier or later) than the *nf-yb2 nf-yb3* background. Thus, we reasoned that we could further ascertain relationships between gene expression/protein accumulation that might be less apparent in the T1 population averages above. Predictably, higher protein accumulation for most NF-YBs was positively correlated with earlier flowering. We confirmed that the stable T3 *p35S*:*NF-YB* lines we used have detectable overexpression using qRT-PCR (S6 Fig). All the lines tested except *p35S*:*NF-YB10* showed high levels of overexpression. We were unable to collect *p35S*:*NF-YB10* plant lines with high protein expression. We also confirmed the presence of each NF-YB protein using microscopy (S6 Fig). The NF-Y has been demonstrated to regulate flowering through direct binding and regulation of *FLOWERING LOCUS T* (*FT)*. Therefore, we expected that the *p35S*:*NF-YB* overexpressors that rescued the late flowering *nf-yb2 nf-yb3* phenotype would have increased *FT* expression. We found that this was indeed true, *p35S*:*NF-YB2*, *p35S*:*NF-YB3*, and *p35S*:*NF-YB7* all had higher levels of *FT* expression compared to the *nf-yb2 nf-yb3* control (Fig 2E).

The NF-Ys are conserved in all eukaryotes and show a high degree of conservation [32]. Therefore, we were also interested in testing if NF-YB subunits from the fungal and animal kingdom would be able to rescue the late flowering *nf-yb2 nf-yb3* phenotype. To test this, we selected the NF-YB subunit from *Drosophila melanogaster* (DmNF-YB) and *Saccharomyces cerevisiae* (ScNF-YB). The results demonstrate that *p35S*:*ScNF-YB* was able partially to rescue the late flowering *nf-yb2 nf-yb3* phenotype whereas *p35S*:*DmNF-YB* led to even later flowering (S7 Fig). This result may indicate that the flanking regions modify the function of the base functional unit.

### The NF-YB Histone Fold Domain (HFD) alone is necessary and sufficient to activate photoperiodic flowering

**Fig 3:**
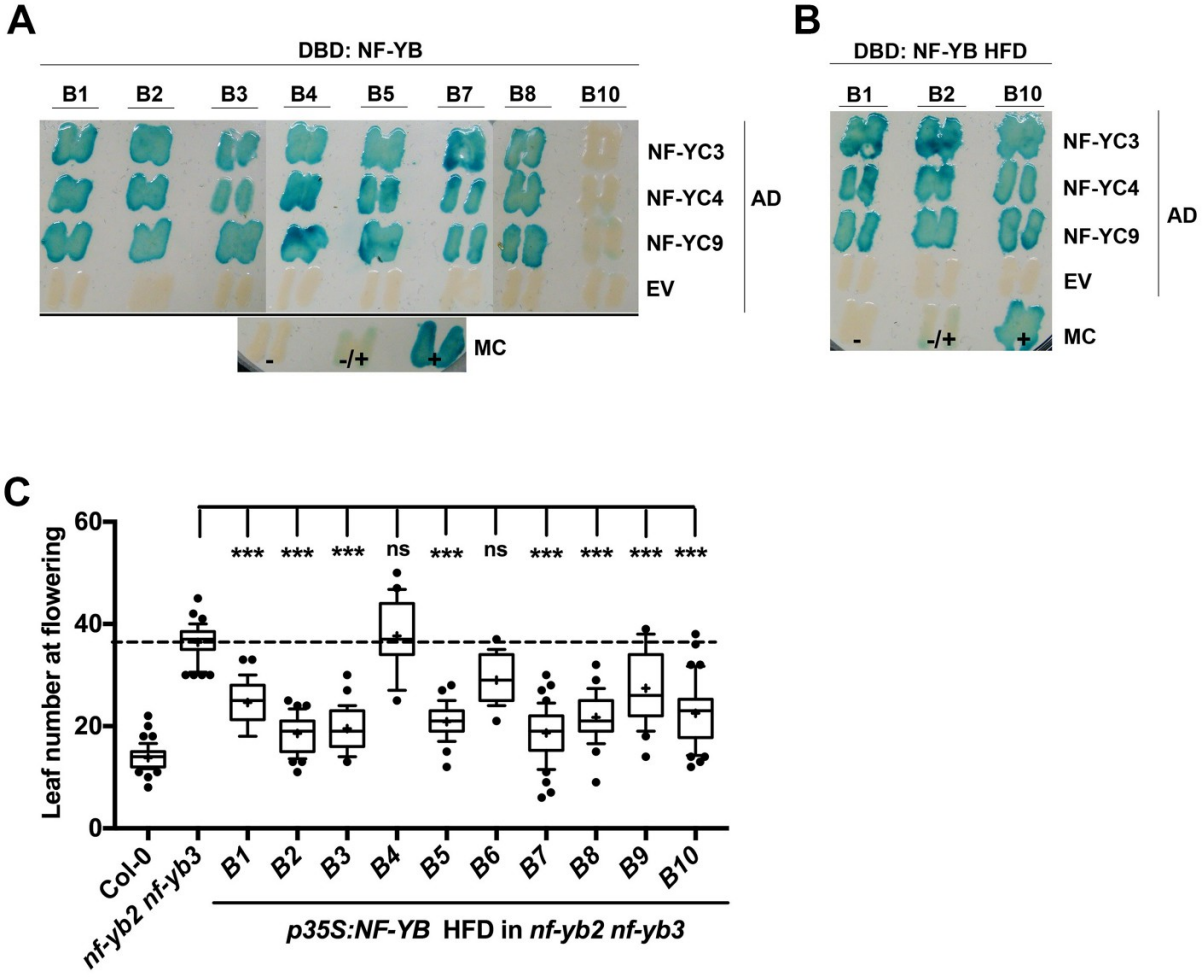
A) Yeast-two hybrid (Y2H) assays testing interactions between selected NF-YBs (full-length protein) and floral-promoting NF-YC3, NF-YC4, and NF-YC9. B) Y2H testing interactions between the Histone Fold Domain (HFD) of selected NF-YBs and floral-promoting NF-YC3, NF-YC4, and NF-YC9. In (A) and (C); DBD: DNA binding domain, AD: activation domain, EV: empty vector control, MC: manufacturer’s controls (- = negative, +/- = intermediate, + = strong positive). C) T1 flowering time quantification of Histone Fold Domain (HFM) of *p35S*::*NF-YB*:*YFP*:*HA* constructs in *nf-yb2 nf-yb3* background. In (C) cross represents mean, outliers represent data points <10th and >90th percentile, respectively. Asterisks represent significant differences derived by the Kruskal-Wallis test (P < 0.05) followed by Dunn’s multiple comparison posthoc tests against Col-0 (A) or *nf-yb2 nf-yb3* (B and C) (* P<0.05, ** P<0.01, *** P<0.001).

Previous studies have demonstrated that the conserved NF-YB HFD in required and sufficient for trimer formation in animal models [39]. Here we used yeast 2-hybrid analysis using selected NF-YB proteins to demonstrate that both the full-length and HFD alone can interact with floral regulating NF-YC subunits, NF-YC3, NF-YC4, and NF-YC9 (Fig 3). As the results demonstrated that the HFD alone can interact with the NF-YC subunits, we were interested in testing if the HFD alone can promote the photoperiod-dependent flowering responses. To test this, we overexpressed each NF-YB HFD in the late flowering *nf-yb2 nf-yb3* mutant. The results demonstrate that most (8/10) of the *p35S*:*NF-YB* HFD can rescue the late flowering phenotype of *nf-yb2 nf-yb3* (Fig 3C).

This finding was notably different from the full-length protein where only three *p35S*:*NF-YB* overexpresses were able to rescue the late flowering *nf-yb2 nf-yb3* phenotype. For example, the full-length *p35S*:*NF-YB1* was not able to rescue the *nf-yb2 nf-yb3* phenotype, however the *p35S*:*NF-YB1* HFD was able to rescue it. These results demonstrated the non-conserved N and C-termini may play a key role in regulating the flowering responses. We further investigated this hypothesis using a domain swap experiment.

### Domain swap experiments between NF-YB1 and NF-YB2 demonstrate that the N- and C-termini have a role in regulating photoperiodic flowering

**Fig 4:**
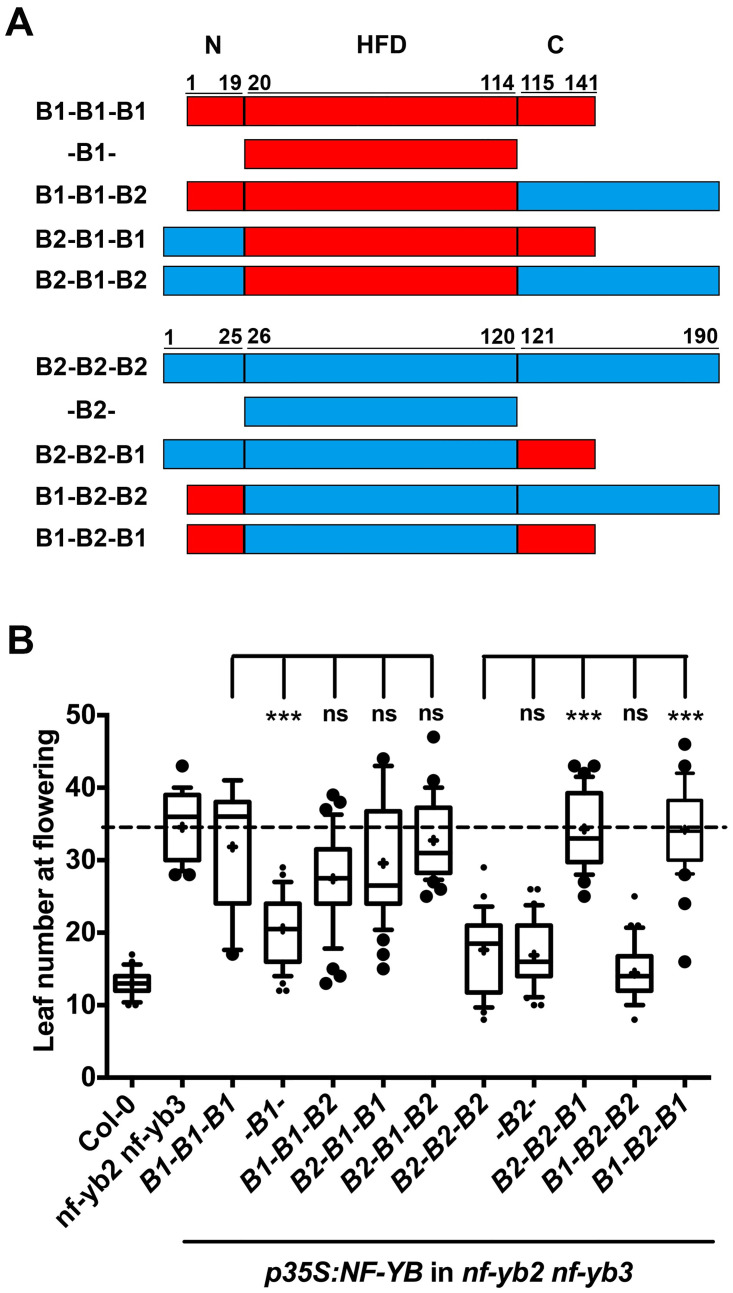
A) Schematic diagram of the *NF-YB1* and *NF-YB2* constructs used in (B). B) T1 flowering time quantification of full-length, Histone Fold Domain (HFD) and domain swaps of *p35S*:*NF-YB1*:*YFP*:*HA* and *p35S*:*NF-YB2*:*YFP*:*HA*. The cross represents mean, outliers represent data points <10th and >90th percentile, respectively. Significance testing was performed by one-way ANOVA (P < 0.05) followed by Dunnett’s multiple comparison post hoc test against *NF-YB1* or *NF-YB2* respectively (* P<0.05, ** P<0.01, *** P<0.001).

We tested the role played by the HFD and the N- and C-termini separately, in regulating photoperiod-dependent flowering by swapping domains between NF-YB1 and NF-YB2 (Fig 4A). Our previous results here demonstrate that the *p35S*:*NF-YB1* full-length protein was not very effective in rescuing the late flowering *nf-yb2 nf-yb3* phenotype whereas *p35S*:*NF-YB1* HFD was able to rescue it. Both *p35S*:*NF-YB2* and *p35S*:*NF-YB2* HFD were able to rescue the late flowering phenotype. We did not notice a dramatic difference in the ability to rescue the late flowering *nf-yb2 nf-yb3* phenotype when attaching the NF-YB1 N-terminus to NF-YB2 or attaching the N- and/or C-termini of NF-YB2 to NF-YB1 (Fig 4B). However, when NF-YB1 C-terminus was attached to NF-YB2, it lost the ability to rescue the *nf-yb2 nf-yb3* late flowering phenotype (Fig 4B). Together with the flowering time responses of *p35S*:*NF-YB* and *p35S*:*NF-YB HFD*, these results demonstrate that the non-conserved N- and C-termini play a key role in regulating floral responses.

### The N- and C-termini of the NF-YB genes may be under pressure for diversifying (positive) selection

**Fig 5:**
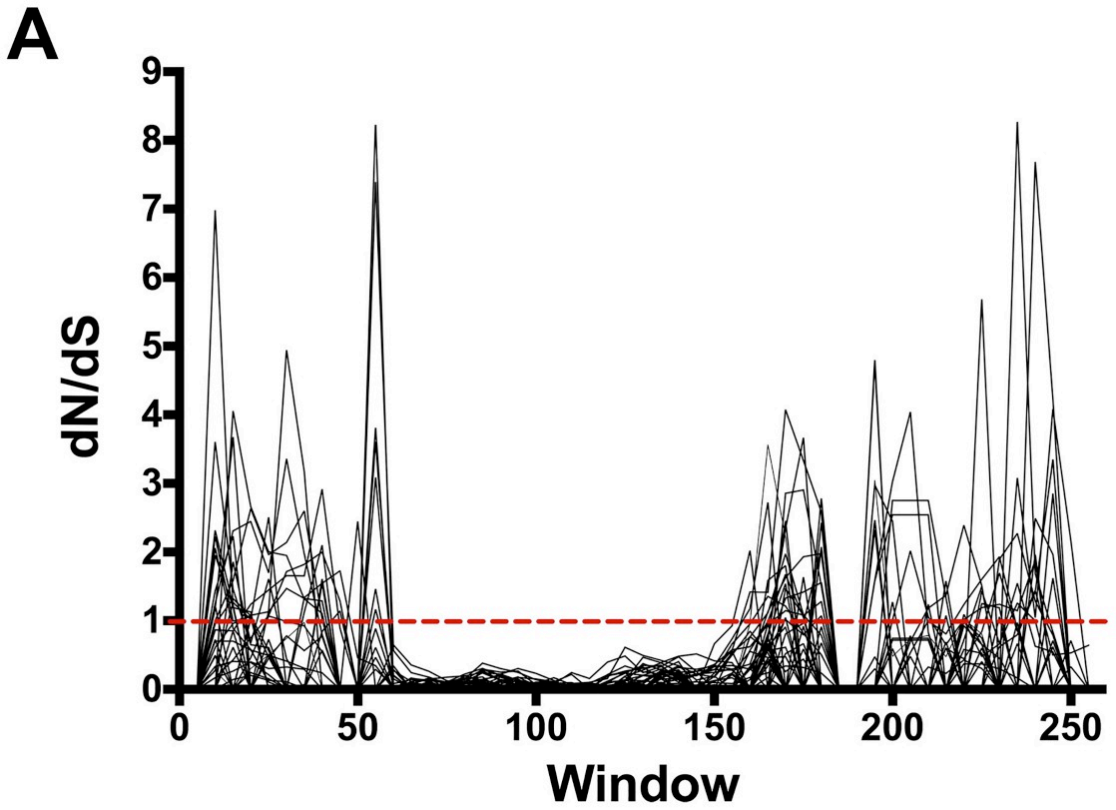
*Ka/Ks* calculations for the NF-YB full-length proteins show that the HFD is under strong purifying selection and the N- and C-termini are under pressure for diversifying selection.

The above results demonstrate that the N- and C-termini of NF-YB genes might have a profound impact on functional specialization. Based on the above results we hypothesized that the HFD, which is required for trimer formation and DNA binding, would be under purifying (negative) selection. Alternatively, the N- and C-termini, which presumably have less selective constraints, would be under pressure for diversifying (positive) selection. To detect purifying/diversifying selection acting on the NF-YB genes we calculated the non-synonymous (*Ka*)/ synonymous (*Ks*) substitution rate between each pair. For the HFD, *Ka/Ks* were consistently below 1 in each pairwise comparison, suggesting that the HFD may be under strong purifying selection (Fig 5). In contrast, the *Ka/Ks* ratio in the N- and C-termini were consistently above 1, indicating that the two termini may be under pressure for diversifying selection. Further in-depth bioinformatics analysis will be conducted to gain precise insights on the impact of selective pressure on the HFD and N- and C-termini.

## Discussion

The main goal in this study was to understand the fate of the duplicated genes in the 10-member Arabidopsis NF-YB gene family. We primarily addressed this question by studying to which extent the NF-YB paralogs can functionally substitute the loss of *nf-yb2 nf-yb3* during the floral promotion. We further extended our study to understand the evolution of protein domains. Multiple sequence alignments confirmed previous findings [22] that all 10 members of the Arabidopsis NF-YB family share a conserved histone fold motif (HFM) flanked by the non-conserved N- and C-termini. The histone fold motif contains amino acids essential to the NF-YB protein-protein interaction and DNA binding. The HFM is highly conserved between the plant, animal, and fungal kingdoms. We expected little divergence in the core amino acids within the HFM and our results supported this observation.

As previously demonstrated in animals [19], we were able to show that the HFM was sufficient for interaction between the NF-YB and NF-YC subunits. This interaction was shown to be functionally significant. Eight of the 10 overexpressors of the HFM were able to rescue the late flowering *nf-yb2 nf-yb3* phenotype. Interestingly, when comparing overexpressors of the HFM with the overexpressors of the full-length proteins, 8/10 HFM overexpressors rescued the late flowering phenotype vs. 3/10 for the full-length protein overexpressors. The non-synonymous (*Ka*)/ synonymous (*Ks*) substitution rate showed that the HFD was under pressure for purifying selection. These results together lead us to conclude that as the NF-YB HFM contains amino acids essential for its basic function and the HFM remains under purifying selection. As most NF-YB HFM can rescue the late flowering *nf-yb2 nf-yb3* phenotype we can assume that the base function (HFM) is required for flowering and the non-conserved N- and C-termini can interfere with that function. This leads to our primary question, what was the fate of the NF-YB genes following gene duplication; how did they change? How did they undergo subfunctionalization and/or neofunctionalization? As the HFM is under purifying selection, we hypothesized that the evolutionary pressure may have been exerted on the N- and C-termini.

Our results support this hypothesis that evolutionary pressure was exerted on the N- and C-termini, which allowed the NF-YB proteins to undergo functional diversification. Multiple sequence alignments demonstrate that the alignment between N and C termini did not show clear positional homology. However, individual alignments of the four subclasses showed that, within a subclass, the N and C termini are more conserved. The conservation within a subclass may explain the observation that members of a subclass tend to share a common biological function, such as NF-YB2, NF-YB3, and NF-YB7 in flowering responses (Class II), and NF-YB6 and NF-YB9 in embryo development (Class IV). Further, the *Ka*/*Ks* substitution rate supported the hypothesis, where results demonstrated that the N- and C-termini of the NF-YB genes may be under pressure for diversifying (positive) selection.

To further test the above possibility, we conducted domain swap experiments. The N- and C-termini of NF-YB1 and NF-YB2 were swapped to observe the flowering response. We chose NF-YB1 for the experiment as it was a weak promoter of flowering responses (on the other hand NF-YB2 was one of the strongest promoters of flowering responses). Attaching the NF-YB2, N- and/or C-termini to NF-YB1 did not lead to a difference in phenotype. While attaching the N-terminus of NF-YB1 to NF-YB2 did not change the phenotype, attaching the C-terminus of NF-YB1 to NF-YB2 lead to the loss of NF-YB2 ability to rescue the *nf-yb2 nf-yb3* late flowering phenotype. This result was interesting as it indicates that the NF-YB1 C-terminus may have diverged and is no longer able to participate in the promotion of flowering time.

In addition to answering questions on gene duplication, our results also demonstrate that the Arabidopsis NF-YB7 protein may be a previously unidentified positive regulator of photoperiod-dependent flowering responses. The previously identified NF-YB subunits that act as positive regulators of photoperiod-dependent flowering are NF-YB2 and NF-YB3 [26]. Interestingly, *p35S*:*NF-YB7* was able to strongly rescue the *nf-yb2 nf-yb3* late flowering phenotype and caused significantly earlier flowering in both the Col-0 and *nf-yb2 nf-yb3* background. NF-YB7 is expressed in the leaves at ~8–12 days and shows the same pattern of expression as NF-YB2 and NF-YB3 indicating a native role in flowering regulation. Therefore, we conclude that here we were able to identify a novel positive regulator of photoperiod-dependent flowering responses, NF-YB7.

In conclusion, this study experimentally demonstrates the fate of a 10-member gene family, the Arabidopsis NF-YB, following gene duplication. Our experimental data demonstrate that while the conserved histone fold domain (HFD) may be under pressure for purifying (negative) selection, the non-conserved N- and C-termini may be under pressure for diversifying (positive) selection. Our data suggest that the non-conserved regions that may be under pressure for diversifying (positive) selection may have undergone functional diversification, which has allowed the retention of the duplicated genes.

## Materials and methods

### Phylogenetic analysis and multiple sequence alignments

Protein and cDNA sequences were obtained for *Arabidopsis thaliana* from The Arabidopsis Information Resource (TAIR) (http://www.arabidopsis.org [40]); *Saccharomyces cerevisiae* from *Saccharomyces* Genome Database (http://www.yeastgenome.org [41]) and *Drosophila melanogaster* from FlyBase (http://www.flybase.org [42]) and manipulated in TextWrangler (http://www.barebones.com). Multiple sequence alignments were made using MUSCLE [43] within Geneious R10 [44]. Phylogenetic analyses were conducted in MEGA7 [45] using full-length protein amino acid sequence, HFD amino acid sequence, and the nucleic acid sequence of the coding region. Phylogenetic trees were estimated using both neighbor-joining (NJ) [46] and maximum likelihood (ML). For ML the best model was determined within MEGA7.

### Cloning and generation of overexpression plant constructs

*p35S*:*NF-YB2* and *p35S*:*NF-YB3* were previously described [26]. All other NF-YB full-length, HFD, and domain swap constructs were amplified from cDNA using the proof-reading enzyme Pfu Ultra II (Agilent Technologies cat#600670–51) and cloned into the Gateway^™^ entry vector pENTR/D-TOPO (Invitrogen, cat#45–0218). All constructs were sequenced and found to be identical to the sequences at The Arabidopsis Information Resource (TAIR) [40]. Plant overexpression constructs were created using the Gateway^™^ LR Clonase II kit (Invitrogen, cat#56485) and subcloned into pEarlyGate101 (ABRC, stock#CD3-683). Plant transformation was done by using the Agrobacterium-mediated floral dip method as previously described [47].

### Plant cultivation and flowering-time experiments

All plants were of the Arabidopsis thaliana Col-0 ecotype and grown at 23°C in standard long day conditions (16-h light/8-h dark), in a custom walk-in growth chamber. Plants were grown in soil containing equal amounts of Farford C2 mix and Metromix 200 supplemented with 40g Marathon pesticide and Peter’s fertilizer. Plants were watered with dilute Peter’s fertilizer (1/10^th^ recommended feeding level). Leaf number at flowering was determined by counting all primary rosette and cauline leaves at bolting.

### RNA extraction and qPCR analysis

Total RNA was collected from 10-day-old seedlings grown in a standard long-day chamber according to the instructions in the E.Z.N.A. Plant RNA Kit (Cat#R6827-01; Omega Biotek). The quality and quantity of RNA samples were confirmed by spectrophotometry (Thermo Scientific, NanoDrop^™^1000). First-strand cDNA synthesis was performed using the Superscript III First-Strand Synthesis System (Invitrogen, cat#18080–051). qPCR was performed using the Bio-Rad CFX96 Real-Time PCR detection System (http://www.bio-rad.com), and the Fermentas Maxima SYBR Green qPCR Master Mix (http://fermantas.com, cat#K0222). Three independent biological replicates were analyzed for each genotype. All samples were normalized to the constitutively expressed gene At2g32170 [48]. Gene expression analysis was performed with the CFX manager software.

### Yeast Two-Hybrid (Y2H) analysis

The LR Clonase II reaction kit (Invitrogen, cat#56485) was used to sub-clone the Gateway^™^ entry clones previously described above in “cloning and generation of overexpression constructs”, into the Gateway compatible ProQuest^™^ Yeast Two-Hybrid system vectors pDEST22 and pDEST32 (Invitrogen, cat#PQ10001-01). X-Gal assays were performed on nitrocellulose membranes containing yeast colonies frozen in liquid nitrogen and incubated at 37°C overnight in Z-buffer containing X-Gal (5-Bromo-4-chloro-3-indoxyl-beta-D-galactopyranoside, Gold Biotechnology, cat#X4281L).

### Protein extraction and western blot

Total protein was extracted from 14-day-old stable plant lines using an improved lysis buffer (20 mM Tris, pH 8.0, 150 mM NaCl, 1 mM EDTA, pH 8.0, 1% Triton X-100, .1% SDS; add fresh 5 mM DTT and 1X Sigma Protease Inhibitor Cocktail (cat #P9599; www.sigmaaldrich.com)). Nuclear fractionation protein preps were performed using a sucrose buffer (20 mM Tris, 0.33M sucrose, 1 mM EDTA; add fresh 5 mM DTT and 1X Sigma Protease Inhibitor Cocktail). Samples were centrifuged at 20XG at 4°C for 30 min and the nuclear pellet was resuspended in an equal volume of sucrose buffer.

Protein samples were separated into 12% polyacrylamide gels. Detection was done using the primary anti-bodies; High-affinity anti-HA (monoclonal 3F10 clone, Roche cat# 11867423001) and custom anti-NF-YC3 (At1g54830) [26] and the secondary anti-bodies; goat anti-rat and goat anti-chicken (Santa Cruz Biotechnology cat# SC-2032, cat#SC-2428). Western blots were visualized on the Bio-Rad ChemiDoc XRS imaging system (www.bio-rad.com) using the horseradish peroxidase-based ECL Plus reagent (GE Healthcare, cat#RPN2132).

### *Ka/Ks* calculation

Positive/negative evolutionary selection was determined using the software tool kit, JCoDA (http://www.tcnj.edu/~nayaklab/jcoda [49]. The unaligned nucleic acid sequence of the full-length-coding region of all 10 Arabidopsis NF-YB genes was uploaded to the program. Alignments were made within JCoDA using ClustalW and Ka/Ks calculations were done using PAML (Phylogenetic Analysis Using Maximum Likelihood, yn00, and codeml). Graphs were made within GraphPad Prism 7 (http://www.graphpad.com).

## Supporting information

S1 Fig Phylogenetic trees of the NF-YB family. Phylogenetic trees were constructed using A) Full-length protein using Maximum-Likelihood (LG Model) with 1000 Bootstrap replicates, B) HFD using Neighbor-Joining with 2000 Bootstrap replicates C) HFD using Maximum-Likelihood (LG Model) with 200 Bootstrap replicates, D) Nucleic acid sequence of the coding region using Neighbor-Joining with 2000 Bootstrap replicates, E) Nucleic acid sequence of the coding region using Maximum-Likelihood (K2 Model) with 200 Bootstrap replicates. All trees were determined and constructed in MEGA7 [50].
(TIF)

S2 Fig Phylogenetic tree of the NF-YB family in *Arabidopsis thaliana*, *Barchypodium distachyon*, *Triticum aestivum*, *Citrus sinensis*, and *Prunus Persia*. The Phylogenetic tree was constructed using full-length protein sequences using Neighbor-Joining with 2000 Bootstrap replicates.
(TIF)

S3 Fig Percent identity and similarity of NF-YB proteins. Percent identity of NF-YB, A) full-length protein B) Histone Fold Domain (HFD). Percent similarity of NF-YB, C) full-length protein D) HFD. Similarity values were calculated using the BLOSM62 matrix. Both identity and similarity matrices were constructed in Geneious. Sc, *Saccharomyces cerevisiae* and Dm, *Drosophila melanogaster*.
(TIF)

S4 Fig Alignment of the NH_2_ (N) termini of the four ancestrally related sub-classes of the Arabidopsis NF-YB. The alignment was constructed using MUSCLE within Geneious.
(TIF)

S5 Fig Alignment of the COOH (C) termini of the four ancestrally related sub-classes of the Arabidopsis NF-YB. The alignment was constructed using MUSCLE within Geneious.
(TIF)

S6 Fig. A) qRT-PCR analysis of NF-YB expression levels of one representative stable T3 generation 35S:NF-YB:YFP:HA construct (line 1 or line 2) in the nf-yb2 nf-yb3 background. B) Localization of NF-YB protein assayed in stable 35S:NF-YB:YFP:HA constructs (TIF)

S7 Fig T1 flowering time quantification of the *Drosophila melanogaster* and *Saccharomyces cerevisiae* (Dm and Sc, respectively) NF-YB subunits in the, A) Col-0 background, and B) *nf-yb2 nf-yb3* background. The cross represents the mean, and outliers represent data points <10th and >90th percentile, respectively. Sample size ≥ 20 independent first-generation transformants. Significance testing was performed by one-way ANOVA (P < 0.05) followed by Dunnett’s multiple comparison post hoc test against Col-0 (A) or *nf-yb2 nf-yb3* (B) (* P<0.05, ** P<0.01, *** P<0.001).
(TIF)

S1 Raw images (PDF)
