# Supplementary figures and images for: Analysis of gene duplication within the Arabidopsis NUCLEAR FACTOR Y, subunit B (NF-YB) protein family reveals domains under both purifying and diversifying selection
Source: PLoS One. 2023 Aug 2;18(8):e0289332. doi: 10.1371/journal.pone.0289332 (PMC10396019; doi:10.1371/journal.pone.0289332)

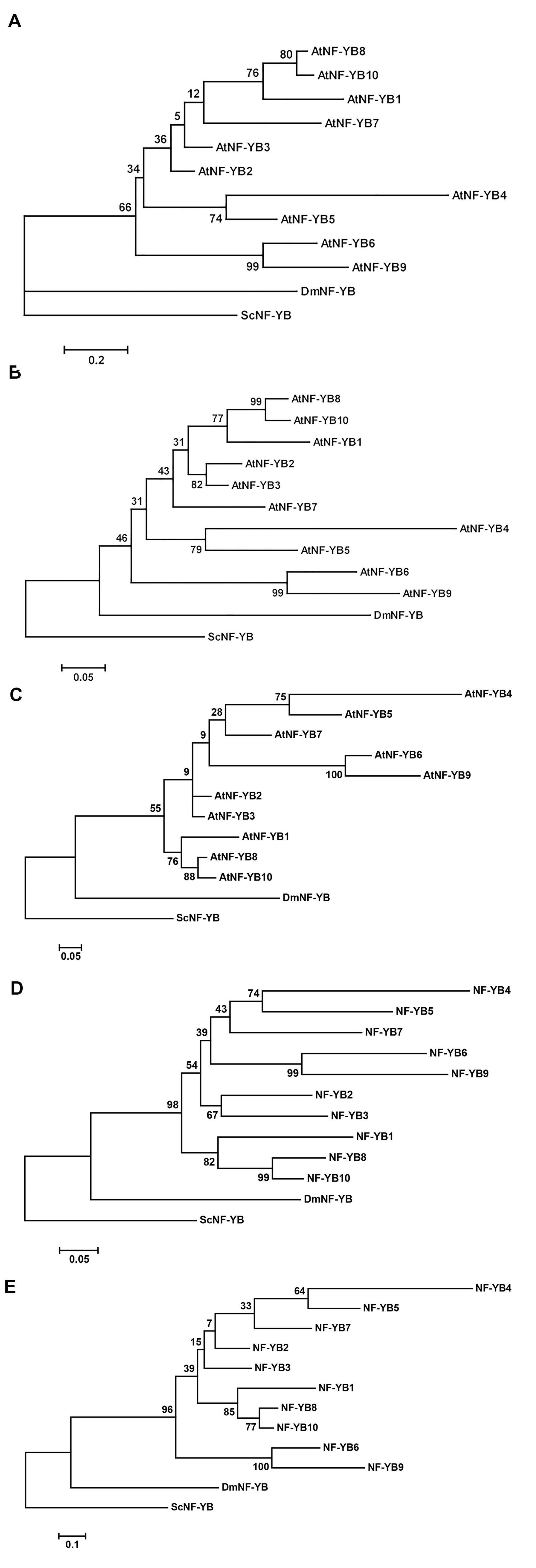

Supplement: S1 Fig — Phylogenetic trees were constructed using A) Full-length protein using Maximum-Likelihood (LG Model) with 1000 Bootstrap replicates, B) HFD using Neighbor-Joining with 2000 Bootstrap replicates C) HFD using Maximum-Likelihood (LG Model) with 200 Bootstrap replicates, D) Nucleic acid sequence of the coding region using Neighbor-Joining with 2000 Bootstrap replicates, E) Nucleic acid sequence of the coding region using Maximum-Likelihood (K2 Model) with 200 Bootstrap replicates. All trees were determined and constructed in MEGA7 [50]. (TIF) [file pone.0289332.s001.tif]

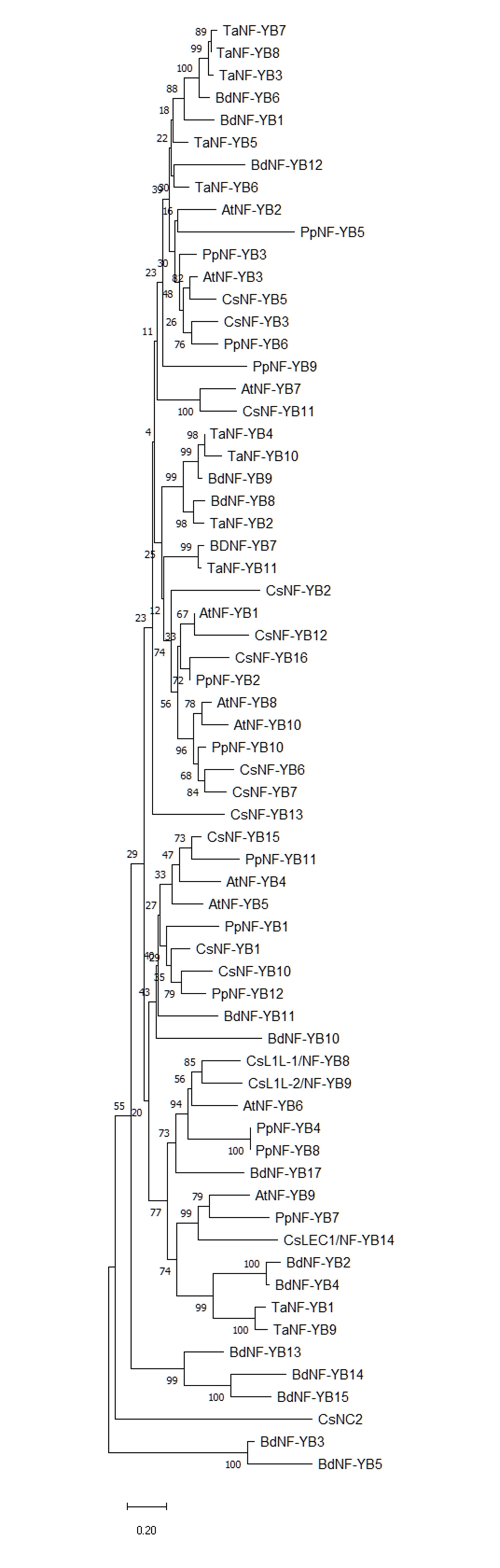

Supplement: S2 Fig — The Phylogenetic tree was constructed using full-length protein sequences using Neighbor-Joining with 2000 Bootstrap replicates. (TIF) [file pone.0289332.s002.tif]

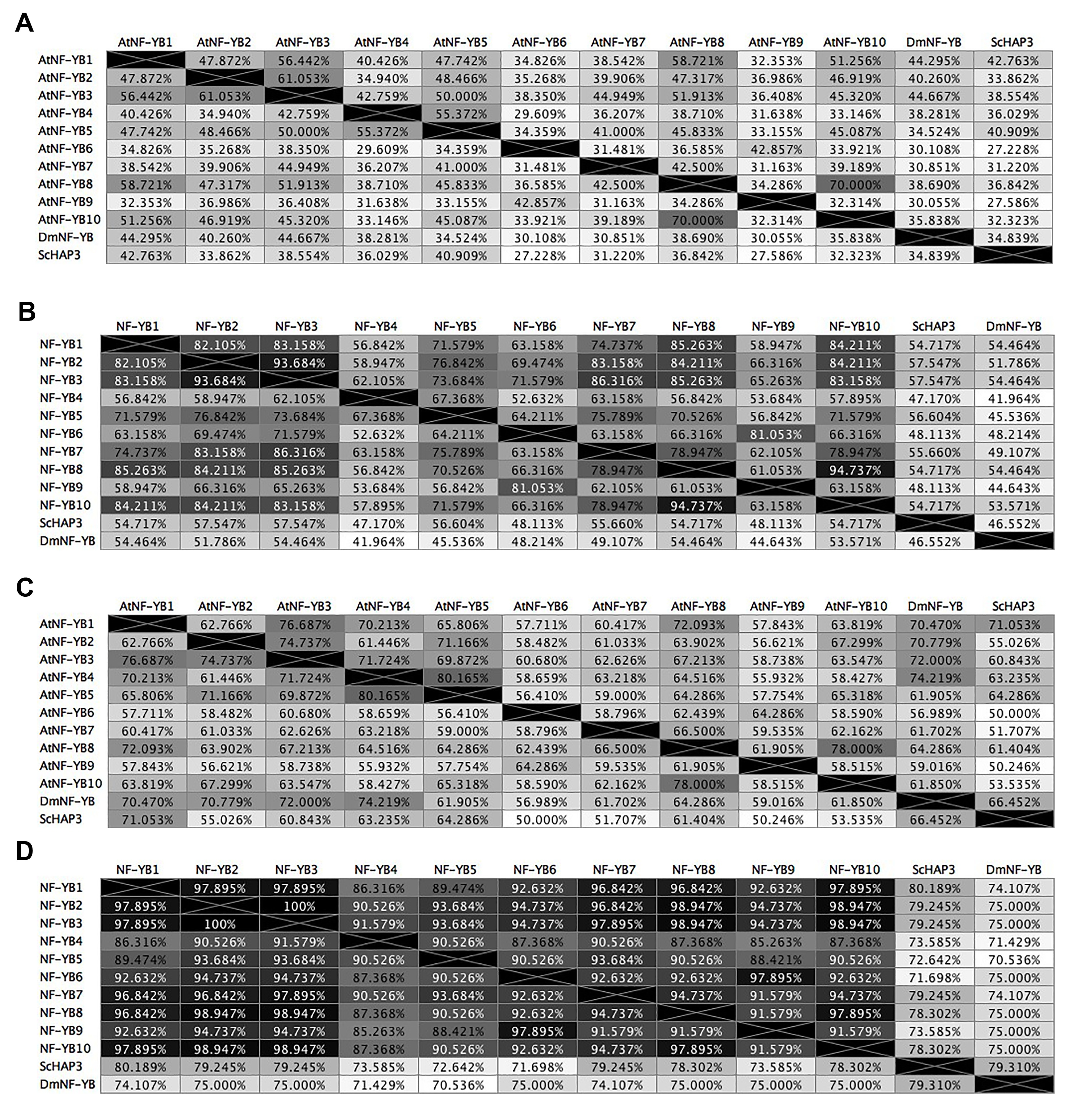

Supplement: S3 Fig — Percent identity and similarity of NF-YB proteins. Percent identity of NF-YB, A) full-length protein B) Histone Fold Domain (HFD). Percent similarity of NF-YB, C) full-length protein D) HFD. Similarity values were calculated using the BLOSM62 matrix. Both identity and similarity matrices were constructed in Geneious. Sc, Saccharomyces cerevisiae and Dm, Drosophila melanogaster. (TIF) [file pone.0289332.s003.tif]

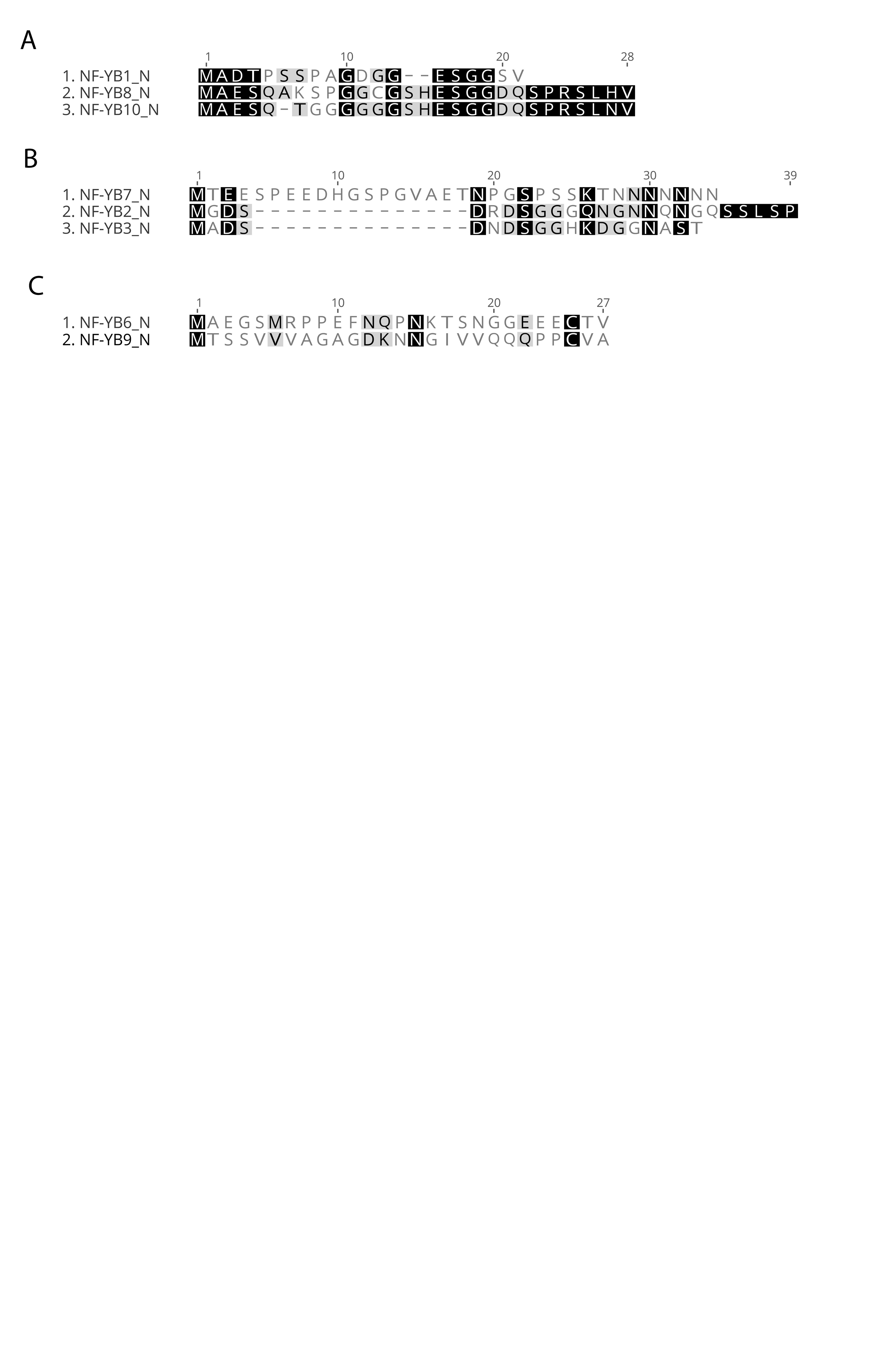

Supplement: S4 Fig — The alignment was constructed using MUSCLE within Geneious. (TIF) [file pone.0289332.s004.tif]

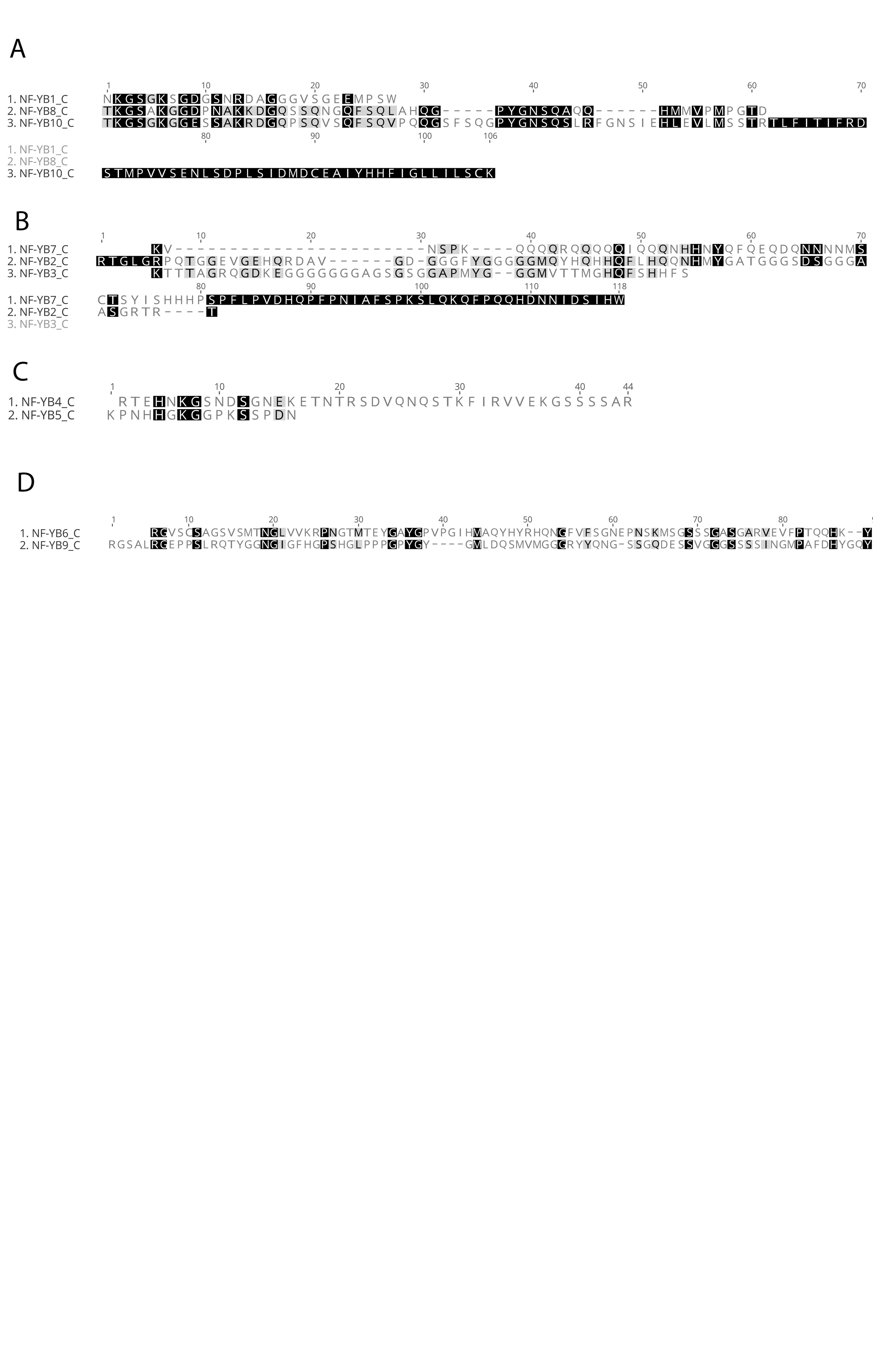

Supplement: S5 Fig — The alignment was constructed using MUSCLE within Geneious. (TIF) [file pone.0289332.s005.tif]

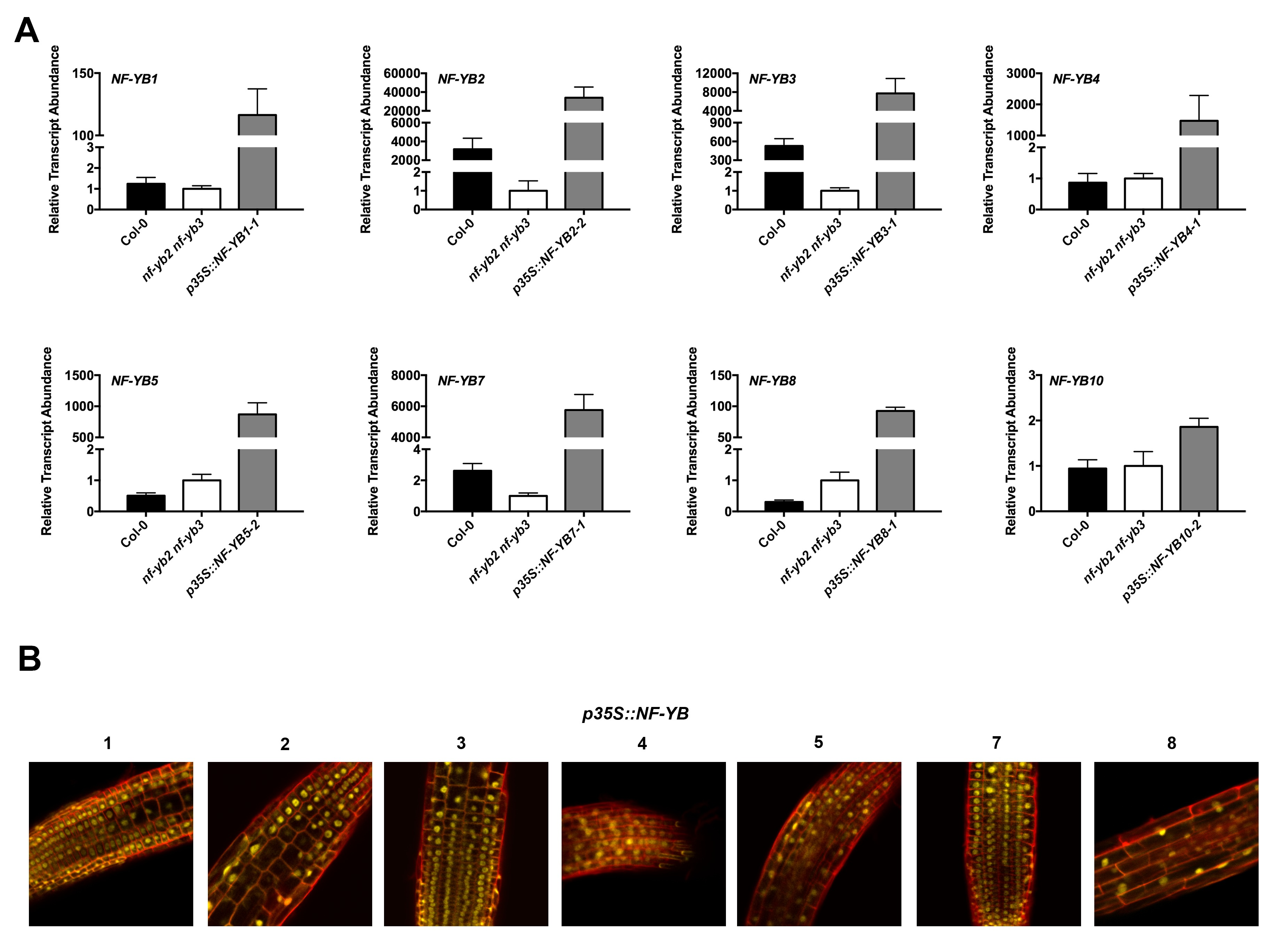

Supplement: S6 Fig. A) qRT-PCR analysis of NF-YB expression levels of one representative stable T3 generation 35S:NF-YB:YFP:HA construct (line 1 or line 2) in the nf-yb2 nf-yb3 background. B) Localization of NF-YB protein assayed in stable 35S:NF-YB:YFP:HA constructs — (TIF) [file pone.0289332.s006.tif]

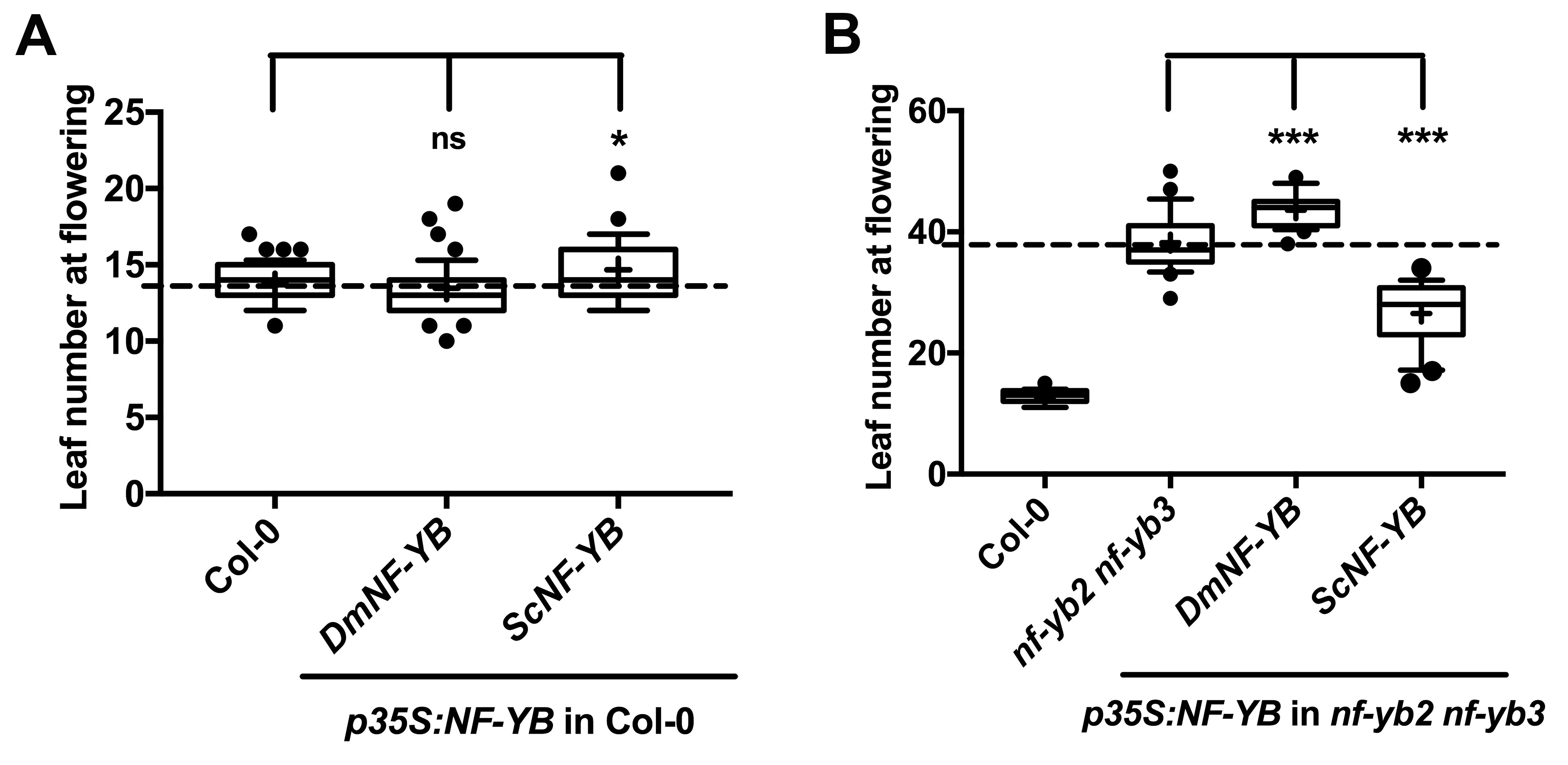

Supplement: S7 Fig — The cross represents the mean, and outliers represent data points <10th and >90th percentile, respectively. Sample size ≥ 20 independent first-generation transformants. Significance testing was performed by one-way ANOVA (P < 0.05) followed by Dunnett’s multiple comparison post hoc test against Col-0 (A) or nf-yb2 nf-yb3 (B) (* P<0.05, ** P<0.01, *** P<0.001). (TIF) [file pone.0289332.s007.tif]
