# Supplementary material for: Analysis of gene duplication within the Arabidopsis NUCLEAR FACTOR Y, subunit B (NF-YB) protein family reveals domains under both purifying and diversifying selection
Source: PLoS One. 2023 Aug 2;18(8):e0289332. doi: 10.1371/journal.pone.0289332 (PMC10396019; doi:10.1371/journal.pone.0289332)
Supplement: S1 Raw images — (PDF) [file pone.0289332.s008.pdf]

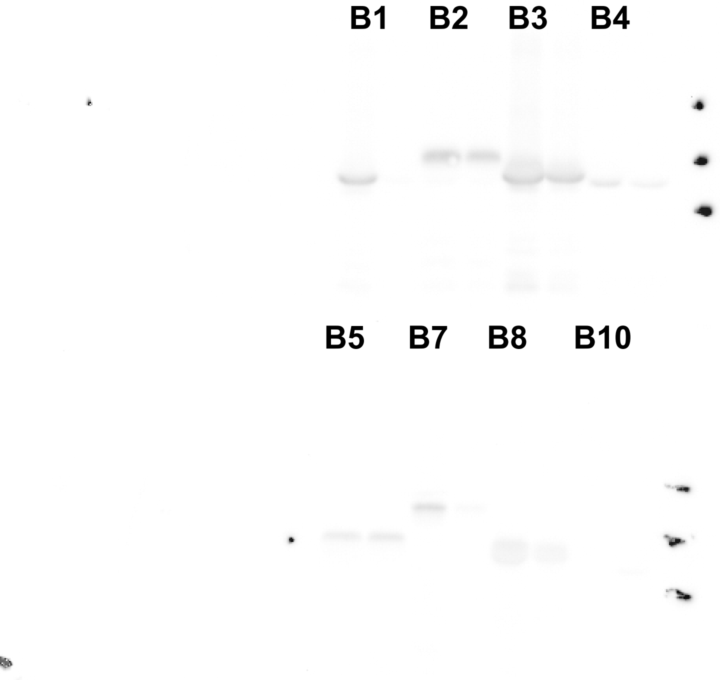

Original image for Fig 2D. The Western blot was visualized on the Bio-Rad ChemiDoc XRS imaging system.
